# On the Colloidal Behavior of Cellulose Nanocrystals as a Hydrophobization Reagent for Mineral Particles

**DOI:** 10.1021/acs.langmuir.0c03131

**Published:** 2021-02-05

**Authors:** Robert Hartmann, Tommi Rinne, Rodrigo Serna-Guerrero

**Affiliations:** Department of Chemical and Metallurgical Engineering, School of Chemical Engineering, Aalto University, P.O. Box 12200, Aalto 00076, Finland

## Abstract

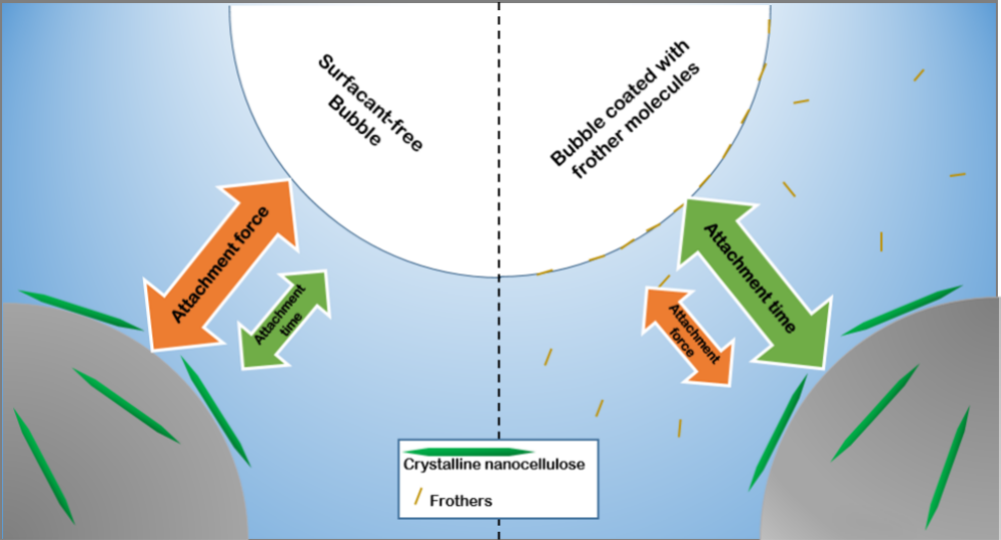

In the search for more sustainable alternatives to the chemical reagents currently used in froth flotation, the present work offers further insights into the behavior of functionalized cellulose nanocrystals as mineral hydrophobization agents. The study corroborates that hexylamine cellulose nanocrystals (HACs) are an efficient collector for the flotation of quartz and also identifies some particular characteristics as a result of their colloidal nature, as opposed to the water-soluble reagents conventionally used. To investigate the individual and collective effects of the frother and HACs on the attachment of particles and air bubbles, an automated contact timer apparatus was used. This induction timer measures particle-bubble attachment probabilities (*P*_att_) without the influence of macroscopic factors present in typical flotation experiments. This allowed the study of the combined influence of nanocellulose and frother concentration on *P*_att_ for the first time. While HACs readily adsorb on quartz modifying its wettability, the presence of a frother leads to a drastic reduction in *P*_att_ up to 70%. The improved recovery of quartz in flotation cells might thus be attributed to froth stabilization by HACs, perhaps acting as a Pickering foam stabilizer. Among the main findings, a tendency of HACs to form mineral agglomerates was identified and further explained using the extended DLVO theory in combination with measured adsorption rates in a quartz crystal microbalance. Therefore, this study distinguishes for the first time the antagonistic effect of frothers on *P*_att_ and their synergies with HACs on the stabilization of orthokinetic froths through the hydrophobization mechanism unlike those of typical water-soluble collectors.

## 1 Introduction

Froth flotation remains the dominant technique for the concentration of a wide variety of minerals. Despite its extensive use, the froth flotation process currently faces increasing challenges, ranging from decreasing ore grades to concerns related to the environmental impact of mining operations.^1,2^ Regarding the latter, one approach that remains relatively unexplored is the use of environmentally friendly reagents in concentrator plants. As it has been recently reported, cellulose derivatives have shown the potential to improve flotation processes as frothers^3−5^ and some examples of high flotation recoveries have been reported with the use of modified nanocellulose collectors for silicates and sulfide minerals.^6−9^ However, it is expected that the use of insoluble reagents with colloidal morphologies introduces fundamentally new features to the hydrophobization mechanisms of mineral surfaces and the performance of flotation processes. While traditional collectors are water-soluble molecules that hydrophobize minerals by selective adsorption, hydrophobization with nanoparticles is based on the formation of stable mineral–nanoparticle aggregates.^10^ This introduces some behavioral complexities, such as the prerequisite to disperse cellulose nanocrystals in aqueous media and maintaining the stability of adsorbed nanoparticles after collision with mineral particles.^11^ Nevertheless, an interesting feature of nanocellulose is that several functional groups may be incorporated onto their surface, allowing for the design of collectors with finely tuned properties.^12−14^ To obtain an optimal collector one should focus on the modification of the surface groups of nanocellulose to reach a balance between mineralophilic and hydrophobic sites, to simultaneously attach on a mineral species while rendering it sufficiently hydrophobic to allow orthokinetic attachments to bubbles, i.e., the formation of permanent aggregates.^15^ This balance of surface groups with different behaviors is exemplified by hexylamine cellulose nanocrystals (HACs), which have showed a promising performance as a collector for quartz in recent studies with recoveries of up to 80 or even 90%, depending on the type of flotation cell and flotation conditions.^7,8,16^ Hence, HACs have shown a high potential to replace water-soluble amine-based collectors, e.g., in reverse flotation of iron ores. Simultaneously, due to its relatively simple chemical composition, quartz was considered as a suitable substrate for further investigations on the behavior of HACs as the hydrophobization reagent.

Due to their complex chemical constitution, it is worth further investigating the potential of nanocellulose-based collectors in flotation, particularly with the use of characterization techniques that help in developing a fundamental understanding of the interactions between bubbles and nanocellulose-modified mineral surfaces. So far, quantitative adsorption of HACs on quartz as a main parameter for evaluating the performance of HACs in flotation processes has not been studied, to our best knowledge. Therefore, a quartz crystal microbalance with dissipation (QCM–D) technique is used in this study to measure the mass of HACs adsorbed on quartz as a function of HAC concentration and time. In addition, the propensity of HACs to adsorb on quartz is quantified using the extended DLVO theory. QCM–D is a highly sensitive in situ surface characterization technique, which has been used recently to study the adsorption of macromolecules,^17^ or in regard to flotation, the adsorption of fatty acid^18^ and nanoparticle collectors.^19^ An advantage of the QCM–D technique is that a similar aqueous condition as used in flotation processes can be mimicked. It is noteworthy that the correlation between the adsorption of collectors measured by QCM-D and flotation performance has not been studied thoroughly. In this work, the focus is on the kinetics of HAC adsorption on quartz, assuming that the spatial dimensions of HACs have an effect on their diffusion onto the mineral surface and thus the required time to render the mineral surface sufficiently hydrophobic.^20^

Furthermore, froth flotation requires the use of a variety of reagents whose behavior in the presence of HACs is yet to be revealed. Among the most common reagents in flotation, the use of frothers is considered a necessity as they are known to adjust bubble size,^21−23^ prevent bubble coalescence and promote either foam stability^24^ or in collaboration with particles froth stability^25^ in flotation processes. It has also been reported that frothers affect the shape and rising velocity of bubbles in a flotation cell, leading to more rigid bubbles and a reduction of their terminal velocity.^26,27^ While the effect of frothers on the dispersion and stability of bubbles is well known, their impact on the particle-bubble attachment probability is still unclear. Indeed, the literature mostly reports on the effects of individual chemical reagents on the particle-bubble interactions, with particular emphasis on collector molecules.^28−36^ The challenge so far has been to find suitable characterization techniques to define the influence of operating conditions on these phenomena, particularly since typical froth flotation experiments in a cell can only report the overall, macroscopic performance of the system.

One may hypothesize that the presence of surfactant molecules at the air–liquid interface has a significant effect on the rupture of the intervening liquid film when a particle approaches, particularly in cases where particles are coated with collector molecules. In a few studies, the effect of the rupture of the intervening liquid film in the presence of frothers and traditional collector molecules was investigated in terms of induction time,^37^ surface tension, thin film thickness and flotation recoveries for different frother and collector concentrations.^38^ For example, Usui and Barouch^35^ theoretically examined the effect of frothers on the van der Waals interactions between bubbles and collector-coated mineral surfaces. They concluded that the polar moieties oriented toward the aqueous phase at the frother-coated interface have a stronger effect on the stabilization of the intervening liquid film than the hydrocarbon chains of amphiphilic collector molecules adsorbed on the solid. In terms of the effect of frothers on the rupture of the intervening liquid film between a bubble and a flat solid surface, Kosior et al.^39^ showed that the roughness of the solid surface has a significant effect on the kinetics of the rupture. Furthermore, while the terminal velocity was reduced with increasing frother concentration, the time for the rupture of the intervening liquid film was prolonged. This additional stability was explained through the presence of nano- or microbubbles on the solid surface, especially in graves and cavities, which were also coated with frother molecules leading to the formation of symmetric foam films between bubbles and flat solid surfaces. Recently, our research group studied the influence of HACs on the floatability of quartz by monitoring fundamental particle-bubble attachments with an automated contact timer apparatus (ACTA) and their correlation with recovery in bench-scale flotation.^16^ It was found that HACs increase the bubble-particle attachment probability and the recovery of quartz in a flotation cell, upholding its effectiveness as a hydrophobization agent. Unlike the characterization techniques used by the other authors, bubbles in the ACTA are formed on a needle tip and subsequently approach a particle bed, consisting of micrometer-scale particles, during a controlled time. Another technique currently attracting attention is based on the attachment of particles on a stationary bubble by either dropping particles from above^40^ or dispersing them in a pulp surrounding the bubble.^41^ However, it should be noted that in these techniques there is an arbitrary kinetic force imposed by the moving particles acting on the rupture of the intervening liquid film between particles and bubbles. In addition, neither the time at the closest approach nor the dimension of the intervening liquid film during the approach or rupture are precisely monitored. On the other hand, ACTA measures the probability of attachment as a function of the relative position of bubbles and the particle bed and contact time, while monitoring the speed of bubbles during approach and retraction. A particularly relevant feature for the objectives of this work is that ACTA is capable of minimizing hydrodynamic factors during the contact event, thus allowing us to evaluate and quantify solely the effect of interfacial interactions between particles and bubbles. Simultaneously, the chemical conditions expected in the flotation pulp can be mimicked. This work thus presents the first ACTA study on the simultaneous influence of collector and frother concentration on the probability of stable particle-bubble attachment events, which is the fundamental requirement in a froth flotation process. As will be detailed in the following sections, through the combination of experimental methods used, it was possible to identify some particularities on the behavior of HACs resulting from their colloidal nature.

## 2 Materials

A 0.1 wt % suspension of HACs was produced following the modification route described by Visanko et al.^42^ The mean diameter of the nanocrystals used here is 5 nm, mean length 137 nm, and amine content 0.76 mol/g.^43^ Based on the size of HACs, the projected area diameter (*d*_a_ = [4*A*/π]^0.5^) of HACs is 29.5 nm, which was used as a characteristic diameter for calculations in the following sections. The nanocrystal sizes were measured via transmission electron microscopy (TEM) and the nitrogen (N^–^) content was measured using a PerkinElmer CHNS/O 2400 Series II elemental analyzer, as reported in a previous work.^43^ To produce HAC suspensions, 10 mM NaCl (Acros Organics, 99 + %) was added to Milli-Q water with the pH value controlled to 7 (± 0.1) and kept constant for 24 h. Under these conditions, the electrostatic attractions between quartz and HACs were shown to be suitable to promote the physisorption of HACs on quartz surfaces in previous studies.^8,16^ Afterward, the initial 0.1 wt % HAC suspension was treated in an ultrasound bath at 40 kHz (Branson 5510 Ultrasonic Cleaner) for 10 min before adding the suspension to the pH 7 background solution in a quantity sufficient for obtaining the target HAC concentration in each experiment. The diluted HAC suspensions were again ultrasonicated for 10 min immediately before each experiment was started to promote an adequate dispersion of the nanocrystals.

**Figure 1:**
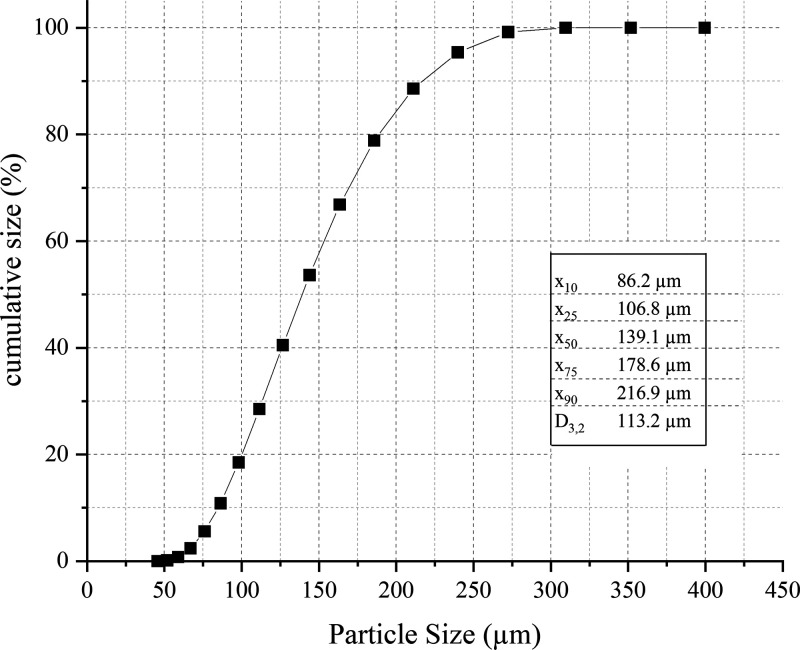
Cumulative particle size distribution of the quartz fraction with different size quantiles and the Sauter mean diameter (D_3,2_).

Quartz was obtained from Nilsiä (Sibelco, Nilsiä, Finland, 100–600 μm) with a nominal purity of 99.2%. For the purposes of this work, a size fraction for ACTA and flotation experiments was prepared as reported earlier.^16^ In short, about 100 g of quartz was ground for 10 s in a ring mill (Fritsch PULVERISETTE, planetary micro mill) made of tungsten carbide. It is expected that no contamination of samples results from grinding. The material was subsequently dry-sieved (Fritsch ANALYSETTE, vibratory sieve shaker) using 75 and 180 μm mesh-opening sizes. The obtained size fraction was dispersed in demineralized water, ultrasonicated for 5 min, placed on a 75 μm mesh-size sieve, and washed with a sufficient amount of demineralized water. After drying, the particle size distribution was measured with a Malvern Mastersizer 3000 and is depicted in Figure 1, along with its measured size quantiles. The removal of fine particles from the mineral sample was performed for the following reasons, based on previously published results:^44^ (i) to prevent the suspended fine particles from reducing the quality of images taken during ACTA measurements, (ii) to prevent a biased estimation of attachment probability due to the spontaneous attachment of nonhydrophobic fine particles on bubbles, and (iii) to minimize the degree of entrainment in flotation experiments.^45^

For the application of the extended DLVO theory, the values of relevant physical parameters of HACs and quartz, such as the surface (ζ-) potential or the surface free energies, were taken from previous studies and are summarized in Table 1.

**Table 1:** Surface (ζ-) Potential and Specific Surface Free Energy Components of HAC and Quartz

	HAC	quartz
surface (ζ-) potential (mV)^8^	13.4	35.2
acidic (γ^+^) surface free energy (mJ/m^2^)^7^	2.4	1.9–8.9
basic (γ^–^) surface free energy (mJ/m^2^)^7^	0.6	4.3–17.7

## 3 Methods

### 3.1 Bench-Scale Froth Flotation

Bench-scale froth flotation experiments were performed in a 1.5 L Outokumpu bench-scale mechanically stirred flotation cell. For each experiment, a pH 7 background solution with 10 mM NaCl was prepared as described above. Approximately 300 g of quartz was dispersed in the background solution and agitated for 5 min with a rotational speed of 1300 rpm. Simultaneously, a mother suspension of HACs was treated for 3 min in an ultrasonic bath and subsequently added to the pulp for 5 min of conditioning at a reduced speed of 600 rpm. Afterward, a frother was added to the solution to obtain concentrations of 5, 10, or 15 ppm and the resulting pulp was further conditioned for 1 min. When the conditioning phase was over, the rotational speed was increased to 1300 rpm and with the introduction of an air flow of 4 L/min, the flotation experiment was started. The total flotation time was 10 min and the froth was collected manually every 30 s. To maintain a constant pulp level in the flotation cell during each experiment, additional background solution including a frother was added from the top of the froth after each sample collection. Due to the periodical collection of the froth via the lip of the flotation cell, the analysis of the froth depth during the experiment was not possible with the setup used here. Admittedly, the measurement of the froth depth with this system calls the attention for future studies. After each experiment, the over- and underflow fractions were collected, vacuum-filtered, and dried in an oven overnight. Subsequently, the mass of the dry products was measured. Due to the relatively large number of experiments carried out, only select experiments were repeated to assess reproducibility.

### 3.2 Quartz Crystal Microbalance

The adsorption of HACs on quartz was quantified with a quartz crystal microbalance with a dissipation unit (QCM–D, Q–Sense Analyzer, Biolin Scientific, Sweden). A pH 7 background solution with 10 mM NaCl was degassed in an ultrasound bath for 10 min and afterward used to condition the quartz sensors at a flow rate of 100 μL/min for 1 h. During conditioning, HAC suspensions with various concentrations were treated in an ultrasound bath for 10 min to avoid the presence of agglomerates and were subsequently introduced at the same flow rate for 30 min. The target concentrations of HACs were 1, 3, 4, 5, 25, or 100 mg/L. The concentration of HAC suspension during the adsorption experiments can be regarded as constant since fresh HAC suspension continuously flowed over the QCM-D crystal. The experiments were performed at 25 °C. To examine whether frother molecules adsorb on the quartz surface, a 100 ppm DF250 sample was prepared as background solution and a QCM–D experiment performed at 25 °C using an identical procedure as described above. Between experiments, a thorough cleaning of the system was performed by heating the sensors up to 60 °C under flowing Milli-Q water at a rate of 100 μL/min. When 60 °C was reached, the flow rate was increased to 300 μL/min and a 2 wt % solution of sodium dodecyl sulfate (SDS, Sigma-Aldrich, ≥99%) was introduced for 5 min followed by 1 h of rinsing with Milli-Q water. Then, the temperature was decreased to 20 °C, and the sensors were removed from the QCM–D and submerged in a pH 3 solution and placed into an ultrasound bath for 30 min with simultaneous heating to 60 °C. Finally, the sensors were rinsed with a sufficient amount of Milli-Q water and dried with nitrogen gas. All experiments were performed as duplicates. To calculate the adsorbed mass of HACs on the quartz sensor, the Sauerbrey equation has been applied^46^
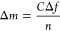
1where Δ*m* is the mass of adsorbed HACs per unit surface, *n* is the overtone number used (in the present case *n* = 3), *C* is the Sauerbrey constant (17.7 ng/[cm^2^s]), and Δ*f* is the detected frequency change.

### 3.3 Automated Contact Timer Apparatus

**Figure 2:**
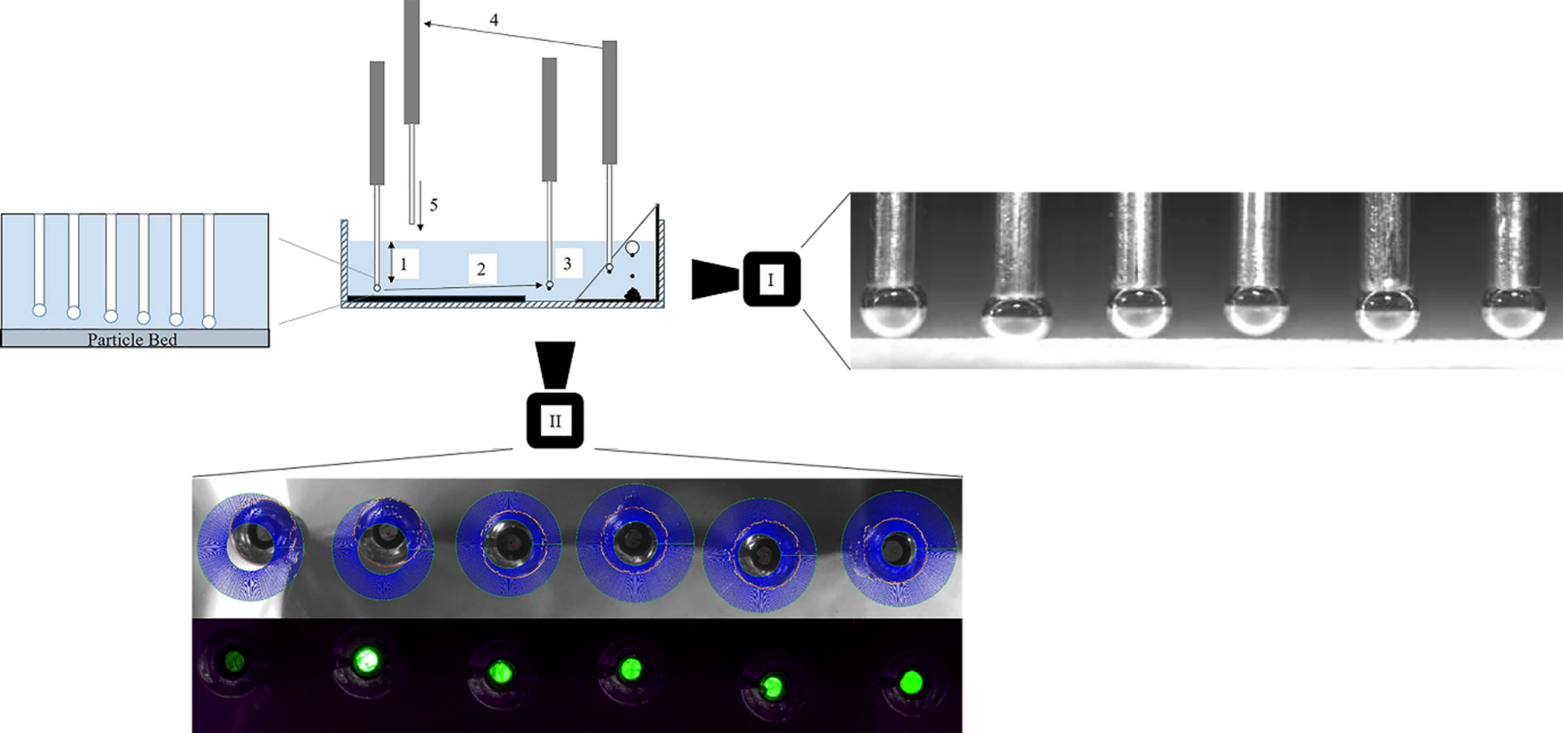
Operating diagram of the ACTA with typical steps of a measurement cycle.^16^

The attachment probability of quartz particles to air bubbles as a function of HAC and frother concentrations was measured with an ACTA. The design and operation of the ACTA has been detailed in previous studies^16,44,47^ and is presented schematically in Figure 2. In brief, the ACTA is a bubble-particle contact timer operating automatically, thus allowing the execution of hundreds of contact events within a short period of time. During operation, ACTA also monitors various relevant aspects of the process, including bubble size, contact time, and approach and recede velocities. A typical measuring cycle consists of the following steps:

1. Bubbles are formed at the tips of the needles and approach the particle bed, rest for a set contact time and recede back to the initial position. The initial position of the needles varied to obtain different distances (*H*) between bubbles and the particle bed for each contact time (left-hand side inset in Figure 2) and the approach, rest and retraction of the bubbles are recorded with a high-speed camera(I). A representative picture showing the position of the bubbles relative to the particle bed at the closest approach is shown on the right-hand side inset in Figure 2
2. Bubbles are transported to the viewing window where pictures are taken by a digital microscope(II), as indicated by the inset image at the bottom of Figure 2. In this image, the blue rings show the target area on which the image processing software finds bubbles. The bubbles recognized by the software are represented by a red line. If a bubble is not recognized properly, e.g., when no bubble is formed on a needle tip, this particular event is excluded from evaluation.
3. Bubbles are moved to a collection bin and detached from the needles. As the bubbles burst upon reaching the surface, any attached particles are collected for subsequent analysis.
4. The needles are lifted out from the liquid, flushed with air for cleaning, and simultaneously moved back on top of the particle bed.
5. The needles are lowered slowly into the liquid over an untouched spot of the particle bed and the next cycle starts

For each experiment, 10 g of quartz was conditioned in a 10 mM NaCl solution at pH 7 with HACs at a concentration of 30, 40, or 50 g/t and Dowfroth 250 (DF250) at 5, 10, or 15 ppm, used as frother. Contact time was set to 20, 40, 60, 80, or 100 ms and 66 cycles were performed for each experiment leading to a total of 396 individual particle-bubbles contact events. A picture of each bubble was taken to monitor their size and determine the successful attachment of particles. The radius of the bubbles varied between 0.9 and 1 mm with minor variations (confidence intervals <0.02 mm), even when changing frother concentrations.

### 3.4 Analysis of Particle–Particle Interactions Based on the Extended DLVO Theory

In previous studies, the DLVO theory was used to predict attractive and repulsive interactions between quartz and cellulose nanocrystals.^8^ In this work, the extended DLVO theory is used to estimate the tendency of quartz particles to form agglomerates in the presence of HACs and the theoretical amount of HACs on quartz required to withstand deagglomeration. For the calculation of extended DLVO interactions between quartz particles, eq 2 was applied
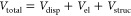
2Accordingly, the total energy of interaction (*V*_total_) represents the sum of macroscopic dispersion (*V*_disp_), electrostatic (*V*_el_) and structural (*V*_struc_) interactions. *V*_disp_ acting between quartz and HAC particles as a function of distance (*H*) was derived from the following equation^48^
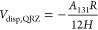
3a
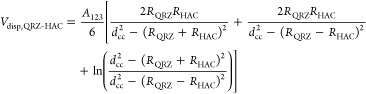
3bHere, *R*_QRZ_ and *R*_HAC_ are the radii of quartz and HACs (*R*_HAC_ = *d*_a_/2), respectively, *d_cc_* is the distance between the centres of the particles, and *A*_123_ is the Hamaker constant. Further, *A*_123_ between quartz and HACs immersed in water is given by^49^
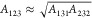
4where *A*_131_ and *A*_232_ are the Hamaker constants, i.e., 50 × 10^–21^ J for quartz^50^ and 8 × 10^–21^ J for HACs^49^ immersed in water. The electrostatic interactions (*V*_el_) are based on the results of electrophoretic mobility tests for quartz and HACs.^8^ The electric surface (ζ-) potential was obtained from the Einstein-Smoluchowski equation^51^
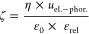
5Here, η is the dynamic viscosity of the aqueous solution at 25 °C, *u*_el. – phor._ is the detected electrophoretic mobility of either quartz or HACs, ε_0_ is the permittivity of a vacuum, and *ε*_rel_ is the relative permittivity of water. With the help of the ζ potential, *V*_el_ between dissimilar spherical particles can be calculated according to eq 6(49)
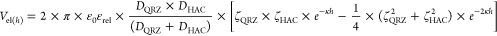
6Here, *D*_QRZ_ and *D*_HAC_ are the diameters of quartz and the projected area diameter (*d*_a_) of HAC and κ is the Debye length. *V*_struc_ between quartz and HACs is given by^48^
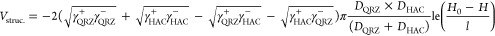
7Here, *l* is the layer thickness of oriented water molecules on the quartz and HAC surface, which was assumed to be 1 nm,^52^*H*_0_ is the minimum separation distance, set to 0.163 nm,^53^ and γ^+^ and γ*^–^* represent nondispersion interactions of quartz and HACs, respectively, and were measured in previous studies using the inverse gas chromatography technique (see Table 1).^7^ The γ^+^ and γ*^–^* values of quartz are functions of the partial coverage of the quartz surface with injected probe molecules and thus both maximum and minimum values of γ*^+^* and γ*^–^* were used for the calculations.

To estimate the potential for particle–particle detachment (*V*_detach_) or the required tenacity for a stable particle–particle agglomeration, the maximum kinetic energy of the particles when the bubbles recede from the particle bed was used as approximation. It is assumed that in a stable bubble-particle agglomerate, the surface forces overcome the corresponding kinetic energy (*E*_kin_), according to eq 8(54)
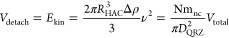
8where Δρ is the difference between the specific weight of water and quartz, being 1000 kg/m^3^ and 2650 kg/m^3^, respectively, and *v* the maximum receding velocity of the needles, obtained from the recording of the automatic movement of the needles, *N* is the number of cellulose nanocrystals, and *m*_nc_ is the mass of a single nanocrystal.

## 4 Results and Discussion

### 4.1 Quartz Flotation

**Figure 3:**
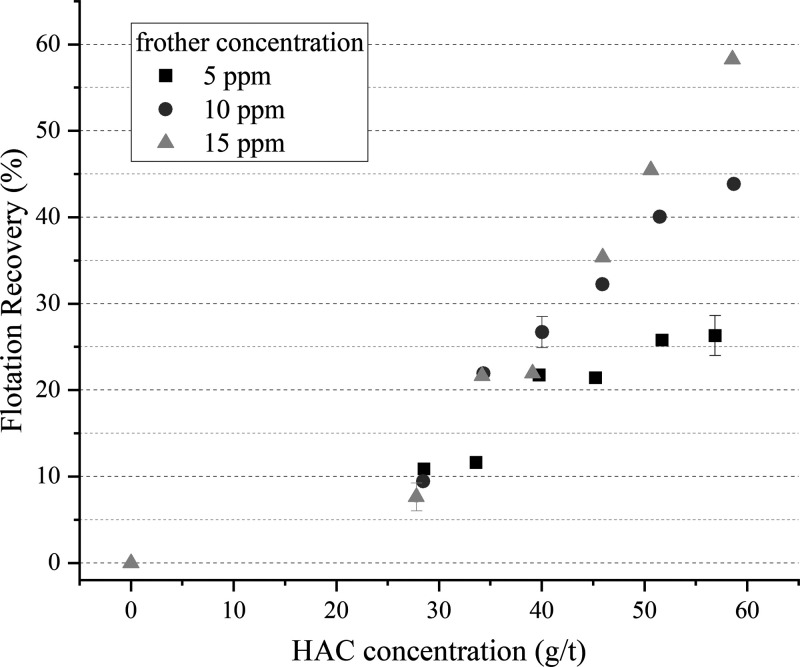
Flotation recovery of quartz as a function of HAC concentration for three different frother concentrations. In the case of experiments performed in duplicate, points represent average values with error bars representing their deviation.

The typical assessment of flotation systems is performed using laboratory-scale flotation experiments which are meant to reproduce the conditions expected in industrial practice and estimate performance in terms of macroscopic parameters such as recovery and grade. It was of interest for this work to carry out, in the first place, flotation experiments to measure the recovery of quartz in the presence of HACs and frothers at various concentrations. The recovery of quartz treated with various concentrations of HACs and floated with the aid of frothers at three different concentrations and are shown in Figure 3.

In the absence of HACs, no quartz was reported to the overflow and only a foam of rapidly collapsing bubbles was formed above the pulp. This may be explained by the absence of hydrophobic particles required for froth formation and stabilization.^25^ Furthermore, the recovery of quartz increased using higher HAC and frother concentrations. At the lowest HAC concentration (i.e., <40 g/ton), the recoveries are similarly low irrespective of the frother concentration used, within experimental error. This may be related to the insufficient hydrophobization of particles, thus resulting in froths of low stability that do not reflect the expected benefits of a higher frother concentration. Nevertheless, as the HAC concentration increases, the overall trends expose higher recovery values using a 15 ppm frother. Thus, the expected promotion of flotation recovery with increasing frother concentration is still regarded as valid. In line with earlier works,^7,8,16^ with increasing HAC concentration, the expected higher degree of hydrophobization of quartz particles lead to improved recoveries. The recovery of quartz also increased with frother concentration, indicating a cooperative effect of the collector and frother reagents. It is worth mentioning that other authors using soluble collectors for quartz flotation studies in a Hallimond tube reported concentrations of 3.7 kg/t for dodecylamine^55^ and 7 kg/t for hexylamine^56^ to achieve recoveries of ca. 80 and 90%, respectively. In a mechanical flotation machine, a concentration of 270 g/t of hexylamine was required for high recoveries of quartz (ca. 90% recovery),^56^ which remains a significantly higher concentration range than that of the HACs used in this study. While the maximum recovery measured in this study is ca. 60%, even recoveries higher than 85% were obtained for a concentration of 50 g/t using a similar HAC sample.^16^ This indicates the first advantage on the use of nanocellulose particles as a collector.

However, the results of a macroscopic flotation experiment tell little about the actual impact of frothers on the interactions between particles and bubbles. According to the well-known Young’s equation, a reduced surface tension at the water–air interface in the presence of frothers leads to the weakening of hydrophobic interactions between an air bubble and a solid particle.^57^ From this fundamental principle, a reduction of the recovery of quartz with increasing frother concentration would be expected but is certainly not observed even when the critical coalescence concentration(CCC)^21,58^ of DF250 (7.2 ppm^59^) is surpassed, i.e., when the Sauter diameter of the bubbles does not decrease with further addition of the frother. Indeed, the contrary is shown in Figure 3, where the well-documented positive effects on the use of frothers are corroborated. Consequently, the effect of frothers on the particle-bubble attachment cannot be evidenced from froth flotation studies alone, as these experiments do not allow the differentiation between interfacial phenomena and macroscopic processes. For this reason, additional characterization using an ACTA was carried out, since its results represent the particle-bubble interactions at a fundamental level as will be discussed in Sections 4.3 and 4.4.

### 4.2 Adsorption Interactions and Kinetics

**Figure 4:**
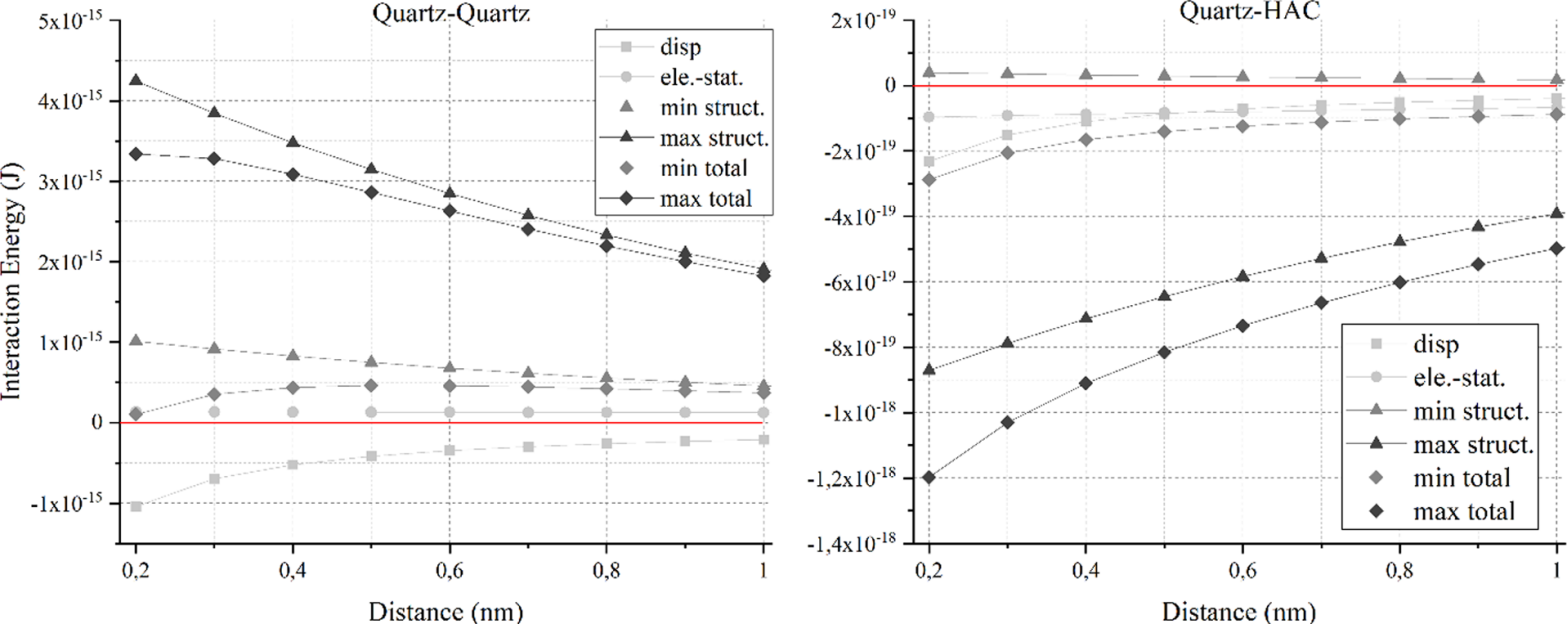
Dispersion (*V*_disp_), electrostatic (*V*_el_), structural (*V*_struct_), and total (*V*_total_) interactions between quartz particles (left) and quartz particles and HACs (right). The red line indicates the zero-level interaction energy and thus the transition from attractive (i.e., IE < 0) to repulsive (i.e., IE > 0) interactions and vice versa.

As a first step to understand the behavior of HACs as a collector, the energy of interaction between quartz particles and HACs was calculated and compared to the interactions between quartz particles using eqs 2–4 and 6−8 with the help of the granulometric properties shown in Figure 1 and the acid–base properties of quartz and HACs given in Table 1. The results are presented in Figure 4 assuming 2 Å as the closest possible distance before the Born repulsion becomes dominant.^60^

In general, pure quartz particles possess predominantly repulsive forces (i.e., IE > 0) between each other. In contrast, the interactions between a quartz particle and HACs are dominantly attractive (i.e., IE < 0), which indicates that HACs are prone to physisorb on the quartz surface. Furthermore, the significance of the specific interactions for both systems was revealed, which are repulsive for equally charged quartz particles and attractive for oppositely charged quartz and HAC particles, dominating the dispersion and electrostatic interactions.

**Figure 5:**
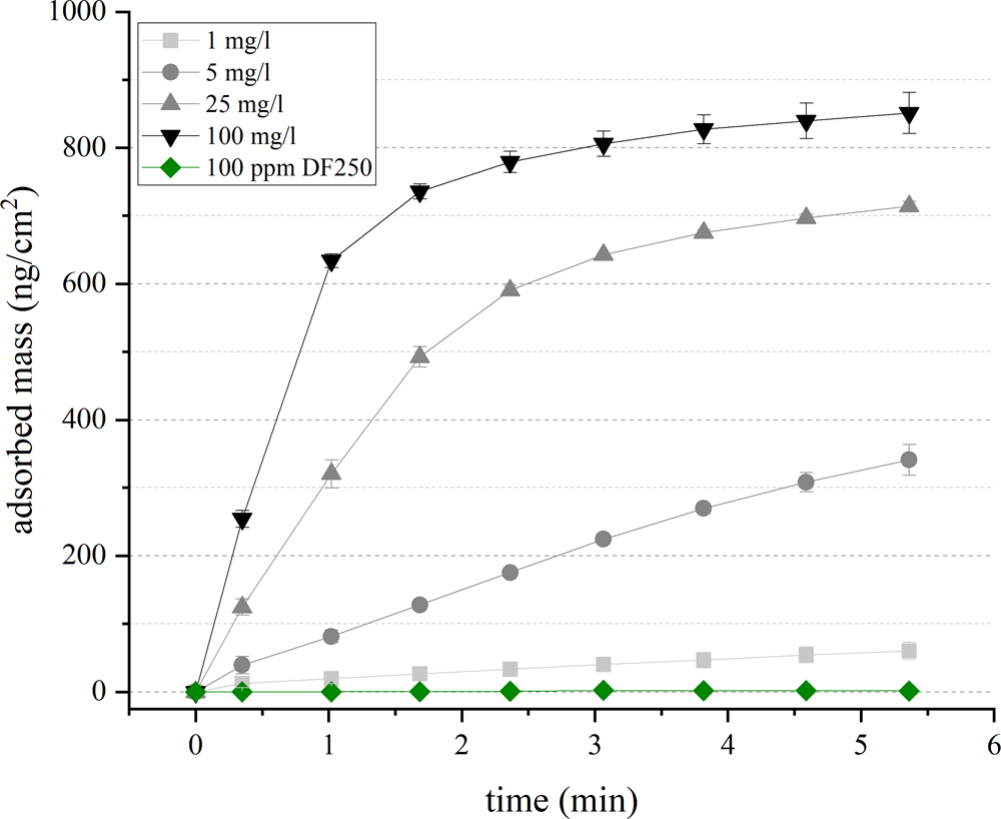
Mass of adsorbed HACs, as a function of HAC concentration, and DF250 over time. Dotted lines represent experimental results and solid lines represent the average.

For the quantification of the adsorbed mass and adsorption kinetics of HACs on a quartz surface, the QCM–D technique was used. The results presented in Figure 5 show the typical trends of physisorption processes where increasing HAC concentration results in higher adsorption capacity and faster adsorption kinetics. For HAC concentrations of 25 and 100 mg/L, the initial adsorption is fast, slowing down after about 1 min to eventually reach a plateau. On the other hand, the adsorption of HACs at low concentrations continues almost linearly for the first 5 min. In consequence, long conditioning times are required to reach equilibrium with HAC concentrations of 5 mg/L or lower. After 5 min, an adsorbed mass of 850 ng/cm^2^ is reached for 100 mg_HAC_/L and only 60 ng/cm^2^ for 1 mg_HAC_/L, indicating that the degree of hydrophobization is a result of HAC concentration and conditioning time. In addition, the experiment with DF250 solution reported no measurable change in the frequency of the QCM detector, meaning that the frother did not adsorb on the bare quartz surface.

To compare the results of the QCM–D with the flotation experiments, the concentration of HACs in the latter was between 5 and 10 mg/L. Although a direct comparison between the adsorption of HACs on quartz during QCM–D experiments and in the flotation pulp is not straightforward, the results obtained by the QCM–D corroborate that a larger mass is adsorbed at higher HAC concentrations within this range. It also shows that adsorption equilibrium was not reached within the conditioning time used. Hence, the flotation performance may be improved by increasing the HAC concentration and by using prolonged conditioning times when possible, although longer conditioning times may require larger vessels or lower throughputs in real industrial practice. In a study by Kou et al.,^61^ the adsorption of dodecylamine (DDA) on a quartz surface was measured as a function of DDA concentration at two different pH values using the QCM-D technique. The adsorbed mass obtained in such study was approximately 40 (at pH 6) to 100 ng/cm^2^ (at pH 9.5) for a DDA concentration of 13 g/L. This indicates that a much greater mass of colloidal HACs adsorbs on quartz surfaces compared to water-soluble DDA under similar conditions. The stronger propensity of HACs to adsorb on quartz correlates well with the higher flotation recoveries achieved with HACs at relatively low concentrations. For a more exact evaluation of the efficiency of water-soluble and colloidal reagents on the hydrophobization of mineral surfaces, a study using both agents under identical conditions will be performed in the future. Nevertheless, the presented results reflect the efficiency of HACs to adsorb on quartz surfaces in comparison with water-soluble collector molecules.^55,56,61^

### 4.3 Influence of HAC and Frother Concentrations on Bubble-Particle Attachment

**Figure 6:**
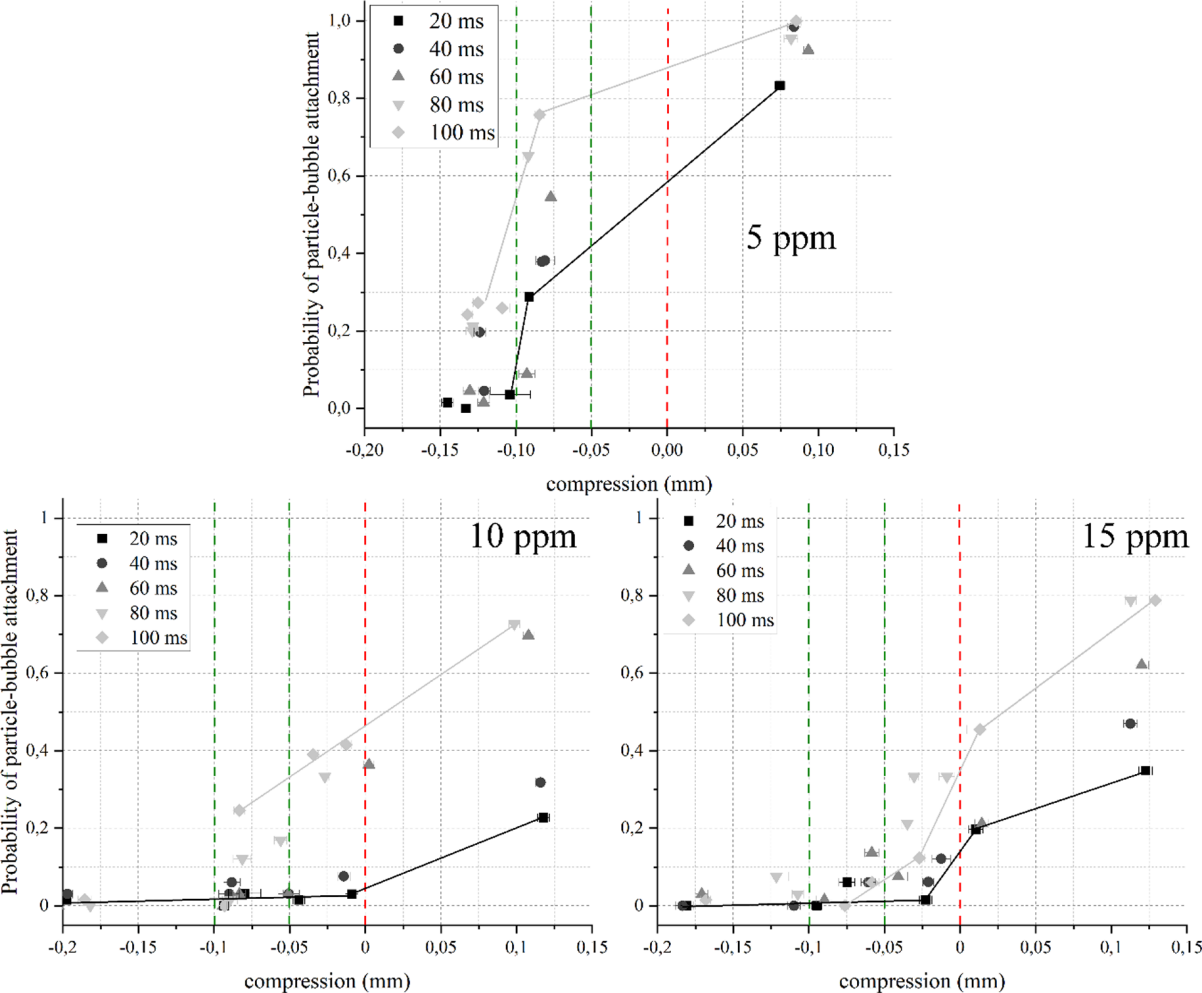
Particle-bubble attachment probability distributions for quartz particles in the presence of 30 g/t HACs and DF250 as a function of contact time and distance (compression = −distance). The red dashed line represents the surface of the particle bed, the green dashed line represents a distance of 800 μm from the particle bed, and black and gray lines are visual guidelines for the shortest and longest contact times. The DF250 concentrations are 5 ppm (up), 10 ppm (left), and 15 ppm (right). Error bars are 95% confidence intervals.

The attachment of a microparticle to an air bubble is the fundamental phenomenon driving separation in true flotation. Given that complex physicochemical processes occur in flotation, a successful particle-bubble attachment can be regarded as a probabilistic event rather than an entirely deterministic one. Figure 6 shows the bubble-particle attachment probability (*P*_att_) distributions as a function of contact time and bubble-particle bed distance (distance = −compression) for quartz modified with 30 g/t of HACs in the presence of various frother concentrations.

The results shown in Figure 6 corroborate the occurrence of noncompressive attachments (i.e., a measurable *P*_att_ at compression <0 mm) for all reagent concentrations, as found for an identical system in the absence of a frother in a previous work.^16^ As expected, *P*_att_ is shown to be sensitive to the contact time between an air bubble and the particle bed, showing higher *P*_att_ for longer contact times. Interestingly, the ACTA results report a significant effect of frother concentration on *P*_att_. Indeed, *P*_att_ was consistently reduced by increasing frother concentration for both noncompressive attachments and attachments under the action of compression. The most striking differences appeared between frother concentrations of 5 and 10 ppm, coincidentally below and above the reported CCC for DF250. Taking the series of 100 ms contact time as an example (gray lines in Figure 6), it is seen that a *P*_att_ of 70% was obtained at a distance of approximately 800 μm with 5 ppm frother concentration. However, by using 10 ppm DF250, the *P*_att_ at this distance drops dramatically down to ca. 25% and close to 0 at 15 ppm. As described in a previous work, lower *P*_att_ or shorter attachment distances are associated with lower hydrophobicity, which in this case can be interpreted as weaker hydrophobic interactions resulting in a stable liquid film between particles and bubbles.^44^ Similar trends were found for HAC concentrations of 40 and 50 g/t, included in the Supporting Information (see Supplementary Material SI and SII).

**Figure 7:**
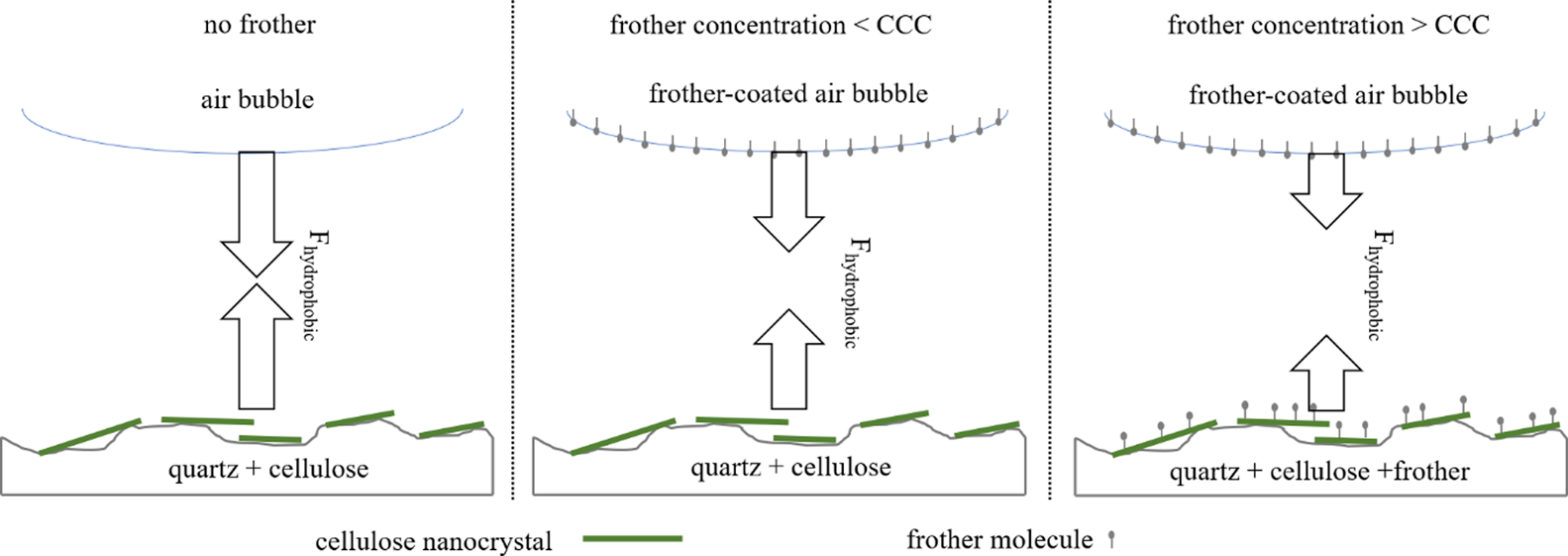
Change in the magnitude of hydrophobic forces acting on the rupture of the intervening liquid film between an air bubble and the quartz surface in the presence of nanocellulose only (left), nanocellulose and a frother at low concentration (middle), and nanocellulose and a frother at high concentration (right).

The results shown in Figure 6 demonstrate the necessity of decoupling the individual phenomena taking place in a flotation process to understand the effect of reagents in microprocesses. Only with a technique such as the one used in the ACTA, it can be demonstrated that with increasing frother concentration and the subsequent decrease of surface tension,^62^*P*_att_ is reduced as predicted by Young’s equation.^57^ In other words, a reduced surface tension leads to a lower hydrophobicity of the bubble surface and thus a weakening of the interactions with the hydrophobic solid surface. The results show that beyond the specific case of HACs and DF250 studied here, the simultaneous action of both reagents on the rupture of the intervening liquid film has to be considered and their chemical design adjusted to an optimum balance between interfacial active and inert or hydrophobic sites. Apart from a thermodynamic explanation for the increased stabilization of the intervening liquid film in the presence of frothers, other authors have proposed phenomenological explanations. For instance, a similar effect of frothers was reported by Kosior et al.,^39^ where a prolongation of the attachment time with increasing frother concentration was detected for bubbles bouncing against a flat solid surface. The delayed rupture of the intervening liquid film was related to the presence of nano- or microbubbles on the solid surface also coated with frother molecules forming a symmetric foam film between the solid surface and a bubble hindering the drainage of the liquid film. In contrast to the work of Kosior, the present study involves for the first time a colloidal system composed of quartz particles hydrophobized with HACs. Interestingly, the effect of an increasing stabilization of the intervening liquid film is obtained even as frother concentrations increased beyond the CCC.^63,64^ This suggests that in addition to the expected adsorption of frother molecules at the gas–liquid interface, frother molecules interact with other available interfaces, likely those on HACs. It is reasonable to suggest that the hydrophobic moieties of frother molecules interact with the hexyl group of HACs, inhibiting their hydrophobization potential. In Figure 7, the various effects of frother molecules on the stabilization of the intervening liquid film for different concentrations is schematically presented.

Consequently, a correlation between the reduction of the surface tension and the solid–liquid interfacial free energy with the stabilization of the intervening liquid film between a bubble and a particle is hereby demonstrated for the first time with the help of the ACTA. Evidently, the stabilization of the intervening liquid film cannot be accounted for using only flotation results, such as those found in Figure 3. The observed increase in the flotation recovery with increasing frother concentration might nevertheless be explained by the positive effect of frothers on bubble size and froth stability prevailing over the antagonistic effects related to the stability of the intervening liquid film. In addition, biopolymer-based particles are well known to efficiently stabilize emulsions and froths due to their heterogeneous surface wettability.^65,66^ Especially shape-anisotropic particles have demonstrated to stabilize emulsions and froths against creaming, coalescence, and drainage at low concentrations as a result of the inter-macromolecular structures and entanglements formed between the fibers.^67,68^ It is thus reasonable to consider that HAC nanoparticles with inhibited hydrophobicity, through the interaction with frother molecules, behave as a Pickering foam stabilizer. Although the action of HACs in the froth phase has been reasonably inferred here, it admittedly requires due quantification. Such a study is out of the scope for this paper but will be part of the future work. Nevertheless, the obtained results indicate that colloidal HACs have several positive effects on the overall flotation performance, although following different mechanisms than those identified for water-soluble reagents.

### 4.4 Influence of Particle Agglomeration on Attachment Probability

**Figure 8:**
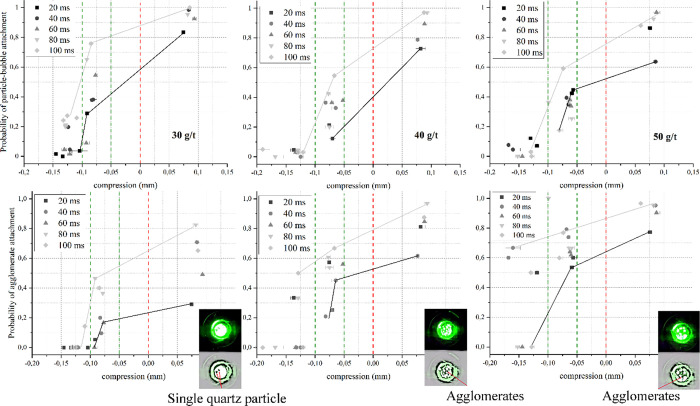
Probability of quartz agglomerates being attached to an air bubble in the presence of HACs at three different concentrations (specified in the graphs) and a DF250 concentration of 5 ppm. Below the probability of agglomerate attachment at identical reagent concentrations with examples of the high contrast image of single particles or agglomerates attached to a bubble during the measurements. Dark and gray lines represent linear trends for the shortest and longest contact time. Error bars are 95% confidence intervals.

The next step of this study was to observe *P*_att_ evolution in the presence of frothers as HAC concentration increases. Figure 8 presents the ACTA results at HAC concentrations of 30, 40, and 50 g/t and 5 ppm DF250. At first glance, these results appear to be in contradiction to an expected increase of *P*_att_ with higher collector concentration at any given particle-bubble distance. For instance, the upper graphs in Figure 8 show that at a distance of approximately 500 μm, the highest *P*_att_ was obtained for the lowest HAC concentration. Similarly, for the case where bubbles were compressed against the particle bed, the lowest HAC concentration leads to the highest *P*_att_. This opposes an expected increase in hydrophobicity at higher collector concentrations. However, a reasonable explanation to this can be found by introducing the concept of probability of agglomeration (*P*_agglo_) for the analysis of results, as shown in the lower graphs in Figure 8. Unlike the commonly used definition of *P*_att_, which only accounts for successful attachment events, *P*_agglo_ is defined as the number of agglomerates being attached to an air bubble related to the total number of successful attachments. For instance, with 30 g/t of HACs, less than 55% of the successful attachment events included agglomerates attached to a bubble at a distance of 500 μm. However, increasing HAC concentration to 40 and 50 g/t leads to *P*_agglo_ values of 70 and 80%, respectively. *P*_agglo_ also increases with HAC concentration when bubbles were compressed against the particle bed. Accordingly, a higher *P*_agglo_ is associated with a greater mass of mineral attached to each bubble, as has been similarly shown recently by October et al.^69^ This shows that the decreasing *P*_att_ reported here with increasing HAC concentration is not related to a lower hydrophobicity but to a greater mass of the attached particles concentrated within a lower number of successful attachment events.^44^

These results demonstrate that the phenomenon of the formation of quartz agglomerates in the presence of HACs has to be taken into consideration for the interpretation of ACTA results and likely for any contact timer technique using particle beds. The formation of particle clusters also reflects the unique functionalization mechanism of HACs, as it is likely a result of the several functional groups available to physisorb on adjacent mineral surfaces and is yet another unique characteristic stemming from its colloidal nature. Similar trends can be seen at higher frother concentrations, as seen in the Supplementary Material SIII (10 ppm) and SIV (15 ppm).

**Figure 9:**
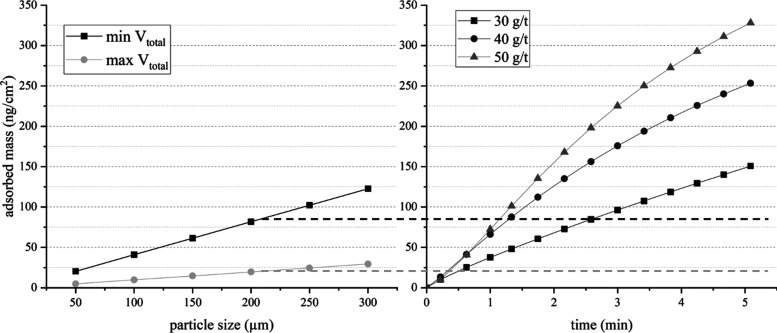
Coverage of quartz with HACs required for stable agglomerates (withstanding maximum kinetic energy) using the minimum (min) and maximum (max) *V*_total_ assuming monolayer formation (left) and results of HACs adsorbed on a quartz sensor using the QCM–D technique. The dotted lines represent the *V*_total_ for particles of the 90% size quantile (see Figure 1).

However, the adsorption of HACs on quartz does not guarantee the formation of stable agglomerates capable of withstanding opposing forces, e.g., when the needles are moved away from the particle bed. For the formation of stable agglomerates, a minimum amount of attractive forces provided by the functional groups in cellulose nanocrystals are required between adjacent quartz particles. With the help of the total interaction energy, the minimum surface coverage by HACs on quartz required to withstand deagglomeration can be derived in accordance to eq 8. The results are shown in Figure 9 and compared to adsorption results using QCM–D. As seen, the amount of HACs adsorbed under the concentrations used in this study is invariably higher than the minimum amount required for stable agglomeration, even under the least favourable conditions of minimum *V*_total_.

Although the results in Figure 9 present only an idealized case, based on the Sauerbrey approach, thus neglecting the effect of energy dissipation^70^ and hydrated layers,^71^ the occurrence of agglomerates seems likely to occur with the HAC concentrations used in this study for all particle sizes. Therefore, the results shown in Figure 8 do not represent a reduced hydrophobicity with increasing HAC concentration but can be explained by the increased tendency of the formation of stable agglomerates.

## 5 Conclusions

By expanding the study of HACs as a collector for quartz in the presence of frothers, the present work identified some unique characteristics of colloidal systems that are distinct from the state-of-the-art water-soluble reagents. First, lab-scale flotation studies showed that HACs recover quartz efficiently at concentrations significantly lower than conventional collectors. Using a quartz crystal microbalance, it was corroborated that the adsorption kinetics are a function of HAC concentration, indicating that sufficiently long conditioning times are required to reach adequate hydrophobization. For a detailed investigation of the action of agents at the three-phase interface, the effect of a frother on the rupture of the intervening liquid film between a particle and a bubble in the presence of HACs was studied. The results with ACTA showed that physisorbed HACs result in high probabilities of noncompressive attachments to air bubbles, a behavior associated with hydrophobic particles.

Furthermore, the influence of frother molecules on the intervening liquid film stability has been identified through a reduction in the attachment probability, an effect that goes unnoticed in flotation experiments. Hence, ACTA has shown that it is capable of detecting the microscopic behavior of frothers, adsorbed on the bubble as well as on HAC particles, in terms of the particle-bubble attachment. A second trend of decreasing *P*_att_ with increasing HAC concentration was obtained, which required a closer examination. Aiming this, the images taken by the ACTA (using microscope II, see Figure 2) were analyzed, showing that higher HAC concentration correlated with an increased probability of agglomerates attached to an air bubble. Hence, the mass of mineral attached to an air bubble increased with increasing HAC concentration, an effect that is overlooked with the common *P*_att_ definition based solely on successful or failed attachment events. This was the first time that the effect of agglomerate formation has been recognized and considered for the evaluation of attachment probabilities using an induction timer.

In summary, the usage of HACs leads to the occurrence of two novel phenomena, which have not been reported for any other collector. The first one is the formation of agglomerate clusters of quartz in the presence of HACs and their effect on the evaluation of results of induction timers. The maximum attraction energy from the extended DLVO theory was used to estimate the required coverage of quartz particles by HACs to withstand deagglomeration forces. These results proved that physisorbed HACs are capable of bridging adjacent quartz particles. The second novel phenomenon concerns the action of colloidal hydrophobic nanocellulose in the presence of a frother. Although the hydrophobicity of HACs may be inhibited by an interaction with surfactants, a stable froth was observed that produced high recoveries of quartz. In contrast to water-soluble agents, stable colloidal HACs are prone to act as a Pickering stabilizer in the froth phase.^65,66^ This potential is solely allocated to colloidal agents and unknown in the field of water-soluble molecules. In consequence, in addition to an efficient hydrophobization of the quartz surface, HACs present in the pulp may improve the overall performance of flotation processes by stabilizing the froth phase and thus efficiently hindering collected particles to drop back into the flotation pulp.

The aforementioned effects, identified through a combination of carefully chosen characterization methods, show that colloidal reagents have distinct interaction mechanisms and adsorption kinetics with mineral surfaces. Such phenomena need dutiful consideration in the interpretation of characterization results and the corresponding design of processes in mineral processing and any other relevant discipline.

## Supporting Information Available

Probability of particle-bubble attachment at 40 g/t and 50 g/t HAC and different DF250 concentrations and probability of particle-bubble and agglomerate attachment at 10 and 15 ppm DF250 and different HAC concentrations (PDF)

## References

[ref1] Bridge G. Contested Terrain: Mining and the Environment. Annu. Rev. Environ. Resour. 2004, 29, 205–259. 10.1146/annurev.energy.28.011503.163434.

[ref2] Calvo G.; Mudd G.; Valero A.; Valero A. Decreasing Ore Grades in Global Metallic Mining: A Theoretical Issue or a Global Reality?. Resources 2016, 5, 36 10.3390/resources5040036.

[ref3] Nuorivaara T.; Serna-Guerrero R. Unlocking the potential of sustainable chemicals in mineral processing: Improving sphalerite flotation using amphiphilic cellulose and frother mixtures. J. Cleaner Prod. 2020, 261, 121143 10.1016/j.jclepro.2020.121143.

[ref4] Nuorivaara T.; Serna-Guerrero R. Amphiphilic cellulose and surfactant mixtures as green frothers in mineral flotation. 1. Characterization of interfacial and foam stabilization properties. Colloids Surf., A 2020, 604, 125297 10.1016/j.colsurfa.2020.125297.

[ref5] Nuorivaara T.; Serna-Guerrero R. Amphiphilic cellulose and surfactant mixtures as green frothers in mineral flotation. 2. Flotation of chalcopyrite and Cu-containing tailings. Colloids Surf., A 2020, 603, 125298 10.1016/j.colsurfa.2020.125298.

[ref6] Laitinen O.; Hartmann R.; Sirviö J. A.; Liimatainen H.; Rudolph M.; Ämmälä A.; Illikainen M. Alkyl aminated nanocelluloses in selective flotation of aluminium oxide and quartz. Chem. Eng. Sci. 2016, 144, 260–266. 10.1016/j.ces.2016.01.052.

[ref7] Hartmann R.; Rudolph M.; Ämmälä A.; Illikainen M. The action of cellulose-based and conventional flotation reagents under dry and wet conditions correlating inverse gas chromatography to microflotation studies. Miner. Eng. 2017, 114, 17–25. 10.1016/j.mineng.2017.09.004.

[ref8] Hartmann R.; Kinnunen P.; Illikainen M. Cellulose-mineral interactions based on the DLVO theory and their correlation with flotability. Miner. Eng. 2018, 122, 44–52. 10.1016/j.mineng.2018.03.023.

[ref9] Lopéz R.; Jordão H.; Hartmann R.; Ämmälä A.; Carvalho M. T. Study of butyl-amine nanocrystal cellulose in the flotation of complex sulphide ores. Colloids Surf., A 2019, 579, 123655 10.1016/j.colsurfa.2019.123655.

[ref10] Abarca C.; Ali M. M.; Pelton R. H. Choosing mineral flotation collectors from large nanoparticle libraries. J. Colloid Interface Sci. 2018, 516, 423–430. 10.1016/j.jcis.2018.01.080. 29408132

[ref11] Dong X.; Price M.; Dai Z.; Xu M.; Pelton R. Mineral-mineral particle collisions during flotation remove adsorbed nanoparticle flotation collectors. J. Colloid Interface Sci. 2017, 504, 178–185. 10.1016/j.jcis.2017.05.050. 28550748

[ref12] Al-Shatty W.; Lord A. M.; Alexander S.; Barron A. R. Tunable Surface Properties of Aluminum Oxide Nanoparticles from Highly Hydrophobic to Highly Hydrophilic. ACS Omega 2017, 2, 2507–2514. 10.1021/acsomega.7b00279. 31457596 PMC6641041

[ref13] Yang S.; Pelton R.; Abarca C.; Dai Z.; Montgomery M.; Xu M.; Bos J.-A. Towards nanoparticle flotation collectors for pentlandite separation. Int. J. Miner. Process. 2013, 123, 137–144. 10.1016/j.minpro.2013.05.007.

[ref14] Yang S.; Pelton R.; Raegen A.; Montgomery M.; Dalnoki-Veress K. Nanoparticle Flotation Collectors: Mechanisms Behind a New Technology. Langmuir 2011, 27, 10438–10446. 10.1021/la2016534. 21790133

[ref15] Ralston J.; Dukhin S. S.; Mishchuk N. A. Wetting film stability and flotation kinetics. Adv. Colloid Interface Sci. 2002, 95, 145–236. 10.1016/S0001-8686(00)00083-X. 11843192

[ref16] Hartmann R.; Serna-Guerrero R. A study on the electric surface potential and hydrophobicity of quartz particles in the presence of hexyl amine cellulose nanocrystals and their correlation to flotation. Front. in Mater. 2020, 7, 1–11. 10.3389/fmats.2020.00053.

[ref17] Saarinen T.; Österberg M.; Laine J. Properties of Cationic Polyelectrolyte Layers Adsorbed on Silica and Cellulose Surfaces Studied by QCM-D—Effect of Polyelectrolyte Charge Density and Molecular Weight. J. Dispersion Sci. Technol. 2009, 30, 969–979. 10.1080/01932690802646488.

[ref18] Kou J.; Tao D.; Xu G. Fatty acid collectors for phosphate flotation and their adsorption behavior using QCM-D. Int. J. Miner. Process. 2010, 95, 1–9. 10.1016/j.minpro.2010.03.001.

[ref19] Yang S.; Pelton R.; Montgomery M.; Cui Y. Nanoparticle flotation collectors III: the role of nanoparticle diameter. ACS Appl. Mater. Interfaces 2012, 4, 4882–4890. 10.1021/am301215h. 22871900

[ref20] Docoslis A.; Wu W.; Giese R. F.; van Oss C. J. Measurements of the kinetic constants of protein adsorption onto silica particles. Colloids Surf., B 1999, 13, 83–104. 10.1016/S0927-7765(98)00111-8.

[ref21] Cho Y. S.; Laskowski J. S. Effect of flotation frothers on bubble size and foam stability. Int. J. Miner. Process. 2002, 64, 69–80. 10.1016/S0301-7516(01)00064-3.

[ref22] Laskowski J. S.; Tlhone T.; Williams P.; Ding K. Fundamental properties of the polyoxypropylene alkyl ether flotation frothers. Int. J. Miner. Process. 2003, 72, 289–299. 10.1016/S0301-7516(03)00105-4.

[ref23] Corona-Arroyo M. A.; López-Valdivieso A.; Laskowski J. S.; Encinas-Oropesa A. Effect of frothers and dodecylamine on bubble size and gas holdup in a downflow column. Miner. Eng. 2015, 81, 109–115. 10.1016/j.mineng.2015.07.023.

[ref24] Grau R. A.; Laskowski J. S. Role of Frothers in Bubble Generation and Coalescence in a Mechanical Flotation Cell. Canad. J. Chem. Engineer. 2006, 84, 170–182.

[ref25] Tan S. N.; Yang Y.; Horn R. G. Thinning of a vertical free-draining aqueous film incorporating colloidal particles. Langmuir 2010, 26, 63–73. 10.1021/la9021118.. 19886631

[ref26] Kracht W.; Finch J. A. Effect of frother on initial bubble shape and velocity. Int. J. Miner. Process. 2010, 94, 115–120. 10.1016/j.minpro.2010.01.003.

[ref27] Sam A.; Gomez C. O.; Finch J. A. Axial velocity profiles of single bubbles in water/frother solutions. Int. J. Miner. Process. 1996, 47, 177–196. 10.1016/0301-7516(95)00088-7.

[ref28] Isrealachvili J. N.; Pashley R. M. Measurement of Hydrophobic Interaction between Two Hydrophobic Surfaces in Aqueous Electrolyte Solutions. J. Colloid Interface Sci. 1984, 98, 500–514. 10.1016/0021-9797(84)90177-2.

[ref29] Parker J. L.; Claesson P. M.; Attard P. Bubbles, cavities, and the long-ranged attraction between hydrophobic surfaces. J. Phys. Chem. 1994, 98, 8468–8480. 10.1021/j100085a029.

[ref30] Ducker W. A.; Xu Z.; Israelachvili J. N. Measurements of Hydrophobic and DLVO Forces in Bubble-Surface Interactions in Aqueous Solutions. Langmuir 1994, 10, 3279–3289. 10.1021/la00021a061.

[ref31] Yoon R.-H.; Flinn D. H.; Rabinovich I. Hydrophobic Interactions between Dissimilar Surfaces. J. Colloid Interface Sci. 1997, 185, 363–370. 10.1006/jcis.1996.4583. 9028890

[ref32] Gillies G.; Kappl M.; Butt H.-J. Direct measurements of particle-bubble interactions. Adv. Colloid Interface Sci. 2005, 114-115, 165–172. 10.1016/j.cis.2004.08.003. 15936290

[ref33] Rudolph M.; Peuker U. A. Hydrophobicity of Minerals Determined by Atomic Force Microscopy - A Tool for Flotation Research. Chem. Ing. Tech. 2014, 86, 865–873. 10.1002/cite.201400017.

[ref34] Preuss M.; Butt H. J. Direct Measurement of Particle–Bubble Interactions in Aqueous Electrolyte: Dependence on Surfactant. Langmuir 1998, 14, 3164–3174. 10.1021/la971349b.

[ref35] Usui S.; Barouch E. Effect of Adsorbed Layers on the van der Waals Interaction between Particles and Bubbles in Aqueous Media. J. Colloid Interface Sci. 1990, 137, 281–288. 10.1016/0021-9797(90)90062-S.

[ref36] Yoon R.-H.; Ravishankar S. A. Long-Range Hydrophobic Forces between Mica Surfaces in Dodecylammonium Chloride Solutions in the Presence of Dodecanol. J. Colloid Interface Sci. 1996, 179, 391–402. 10.1006/jcis.1996.0230.

[ref37] Lekki J.; Laskowski J. On the dynamic effect of frother-collector joint action in flotation- Lekki & ALaskowski. Min. process. & Extra. metal. 1971, 80, 172–180.

[ref38] Manev E.; Pugh R. J. Frother/collector interactions in thin froth films and flotation. Colloids Surf., A 1993, 70, 289–295. 10.1016/0927-7757(93)80302-U.

[ref39] Kosior D.; Zawala J.; Krasowska M.; Malysa K. Influence of n-octanol and α-terpineol on thin film stability and bubble attachment to hydrophobic surface. Phys. chem. chem. phys. : PCCP 2013, 15, 2586–2595. 10.1039/c2cp43545d.. 23322074

[ref40] Arai N.; Watanabe S.; Miyahara M. T.; Yamamoto R.; Hampel U.; Lecrivain G. Direct observation of the attachment behavior of hydrophobic colloidal particles onto a bubble surface. Soft Matter 2020, 16, 695–702. 10.1039/c9sm01787a. 31815273

[ref41] Wang P.; Reyes F.; Cilliers J. J.; Brito-Parada P. R. Evaluation of collector performance at the bubble-particle scale. Miner. Eng. 2020, 147, 106140 10.1016/j.mineng.2019.106140.

[ref42] Visanko M.; Liimatainen H.; Sirviö J. A.; Heiskanen J. P.; Niinimäki J.; Hormi O. Amphiphilic cellulose nanocrystals from acid-free oxidative treatment: physicochemical characteristics and use as an oil-water stabilizer. Biomacromolecules 2014, 15, 2769–2775. 10.1021/bm500628g. 24946006

[ref43] Hartmann R.; Sirviö J. A.; Sliz R.; Laitinen O.; Liimatainen H.; Ämmälä A.; Fabritius T.; Illikainen M. Interactions between aminated cellulose nanocrystals and quartz: Adsorption and wettability studies. Colloids Surf., A 2016, 489, 207–215. 10.1016/j.colsurfa.2015.10.022.

[ref44] Hartmann R.; Serna-Guerrero R. Towards a quantitative analysis of the wettability of microparticles using an automated contact timer apparatus. Miner. Eng. 2020, 149, 106240 10.1016/j.mineng.2020.106240.

[ref45] Smith P. G.; Warren L. J. Entrainment of Particles into Flotation Froths. Miner. Process. Extr. Metall. Rev. 1989, 5, 123–145. 10.1080/08827508908952647.

[ref46] Sauerbrey G. Verwendung von Schwingquarzen zur Wägung dünner Schichten und zur Mikrowägung. Zeitschrift für Physik 1959, 155, 206–222. 10.1007/BF01337937.

[ref47] Aspiala M.; Schreithofer N.; Serna-Guerrero R. Automated contact time apparatus and measurement procedure for bubble-particle interaction analysis. Miner. Eng. 2018, 121, 77–82. 10.1016/j.mineng.2018.02.018.

[ref48] Drzymala J. Mineral Processing:Foundations of theory and practice of minerallurgy, 1st ed.; Drzymala J., 2007.

[ref49] But H.-J.; Kappl M. Surface and Interfacial Forces; WILEY-VCH Verlag GmbH & Co. KGaA, 2010.

[ref50] Ackler H. D.; et al. Comparisons of Hamaker Constants for Ceramic Systems with Intervening Vacuum or Water: From Force Laws and Physical Properties. J. Colloid Interface Sci. 1996, 179, 460–469. 10.1006/jcis.1996.0238.

[ref51] Delgado A. V.; González-Caballero F.; Hunter R. J.; Koopal L. K.; Lyklema J. Measurement and interpretation of electrokinetic phenomena. J. Colloid Interface Sci. 2007, 309, 194–224. 10.1016/j.jcis.2006.12.075. 17368660

[ref52] van Oss C.:J. Interfacial Forces in Aqueous Media; Marcel Dekker, Inc., 1994.

[ref53] Yao J.; Han H.; Hou Y.; Gong E.; Yin W. A Method of Calculating the Interaction Energy between Particles in Minerals Flotation. Mathemat. Prob. in Engineer. 2016, 2016, 1–13. 10.1155/2016/8430745.

[ref54] Yoon R.-H.; Mao L. Application of Extended DLVO Theory, IV. Derivation of Flotation Rate Equation from First Principles. J. Colloid Interface Sci. 1996, 181, 613–626. 10.1006/jcis.1996.0419.

[ref55] Vidyadhar A.; Das A.; Hanumantha Rao K. Adsorption mechanism of long-chain alkylamines on quartz and albite. Inter. Sem. on Mineral Proces Technol. 2008, 306–313.

[ref56] Kowalczuk P. B. Flotation and hydrophobicity of quartz in the presence of hexylamine. Int. J. Miner. Process. 2015, 140, 66–71. 10.1016/j.minpro.2015.05.002.

[ref57] Young T. An Essay on the Cohesion of Fluids. Philosoph.l Trans. R. Soc. of London 1805, 95, 65–87.

[ref58] Cho Y. S.; Laskowski J. S. Bubble coalescence and its effect on dynamic foam stability. Canad. J. Chem. Engineer. 2002, 80, 299–305.

[ref59] Jávor Z.; Schreithofer N.; Heiskanen K. Micro- and nano-scale phenomena effect on bubble size in mechanical flotation cell. Miner. Eng. 2015, 70, 109–118. 10.1016/j.mineng.2014.09.010.

[ref60] Israelachvili J. N. van der Waals dispersion force contribution to works of adhesion and contact angles on the basis of macroscopic theory. J. Chem. Soc., Faraday Trans. 1973, 69, 1729 10.1039/f29736901729.

[ref61] Kou J.; Tao D.; Xu G. A study of adsorption of dodecylamine on quartz surface using quartz crystal microbalance with dissipation. Colloids Surf., A 2010, 368, 75–83. 10.1016/j.colsurfa.2010.07.017.

[ref62] Castro S.; Miranda C.; Toledo P.; Laskowski J. S. Effect of frothers on bubble coalescence and foaming in electrolyte solutions and seawater. Int. J. Miner. Process. 2013, 124, 8–14. 10.1016/j.minpro.2013.07.002.

[ref63] Peng H.; Birkett G. R.; Nguyen A. V. Progress on the Surface Nanobubble Story: What is in the bubble? Why does it exist?. Adv. Colloid Interface Sci. 2015, 222, 573–580. 10.1016/j.cis.2014.09.004. 25267688

[ref64] Azevedo A.; Etchepare R.; Calgaroto S.; Rubio J. Aqueous dispersions of nanobubbles: Generation, properties and features. Miner. Eng. 2016, 94, 29–37. 10.1016/j.mineng.2016.05.001.

[ref65] Lam S.; Velikov K. P.; Velev O. D. Pickering stabilization of foams and emulsions with particles of biological origin. Curr. Opin. Colloid Interface Sci. 2014, 19, 490–500. 10.1016/j.cocis.2014.07.003.

[ref66] Dickinson E. Biopolymer-based particles as stabilizing agents for emulsions and foams. Food Hydrocolloids 2017, 68, 219–231. 10.1016/j.foodhyd.2016.06.024.

[ref67] Alargova R. G.; Warhadpande D. S.; Paunov V. N.; Velev O. D. Foam superstabilization by polymer microrods. Langmuir 2004, 20, 10371–10374. 10.1021/la048647a.. 15544360

[ref68] Kalashnikova I.; Bizot H.; Bertoncini P.; Cathala B.; Capron I. Cellulosic nanorods of various aspect ratios for oil in water Pickering emulsions. Soft Matter 2013, 9, 952–959. 10.1039/C2SM26472B.

[ref69] October L. L.; Corin K. C.; Manono M. S.; Schreithofer N.; Wiese J. G. A fundamental study considering specific ion effects on the attachment of sulfide minerals to air bubbles. Miner. Eng. 2020, 151, 106313 10.1016/j.mineng.2020.106313.

[ref70] Voinova M. V.; Rodahl M.; Jonson M.; Kasemo B. Viscoelastic Acoustic Response of Layered Polymer Films at Fluid-Solid Interfaces: Continuum Mechanics Approach. Phys. Scr. 1999, 59, 391–396. 10.1238/Physica.Regular.059a00391.

[ref71] Liu Z.; Choi H.; Gatenholm P.; Esker A. R. Quartz crystal microbalance with dissipation monitoring and surface plasmon resonance studies of carboxymethyl cellulose adsorption onto regenerated cellulose surfaces. Langmuir 2011, 27, 8718–8728. 10.1021/la200628a.. 21699205

