# Supplementary material for: On the Colloidal Behavior of Cellulose Nanocrystals as a Hydrophobization Reagent for Mineral Particles
Source: Langmuir. 2021 Feb 5;37(7):2322–33. doi: 10.1021/acs.langmuir.0c03131 (PMC8023700; doi:10.1021/acs.langmuir.0c03131)
Supplement: Supplementary file 1 — la0c03131_si_001.pdf [file la0c03131_si_001.pdf]

On the Colloidal Behaviour of Cellulose Nanocrystals as a Hydrophobization Reagent for Mineral Particles

*Robert Hartmann, Tommi Rinne and Rodrigo Serna-Guerrero**

Department of Chemical and Metallurgical Engineering, School of Chemical Engineering, Aalto University, P.O. Box 12200, 00076 Aalto, Finland

*(*corresponding author: rodrigo.serna@aalto.fi)*

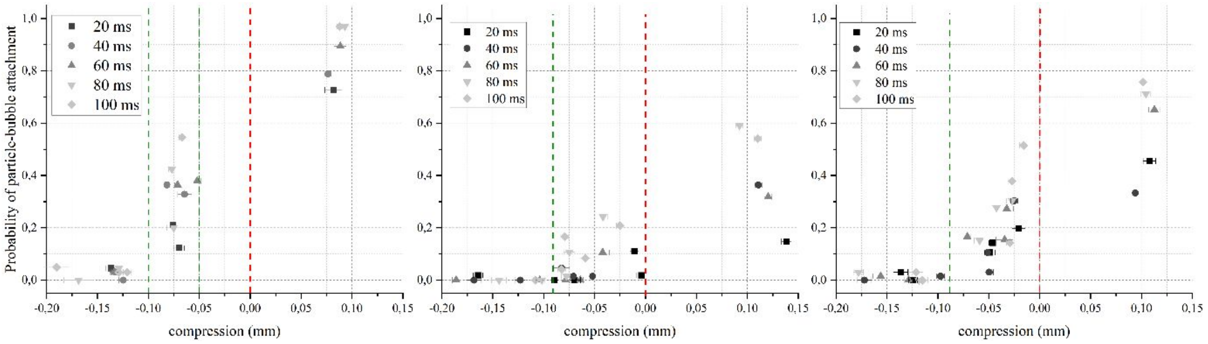

S1: Probability of particle-bubble attachment at 40 g/t HAC and 5 ppm (left), 10 ppm (middle) and 15 ppm DF250.

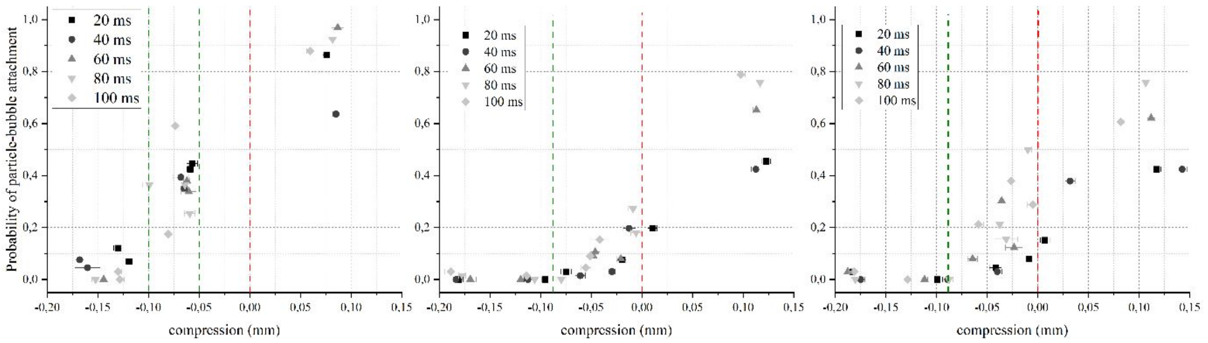

S2: Probability of particle-bubble attachment at 50 g/t HAC and 5 ppm (left), 10 ppm (middle) and 15 ppm DF250.

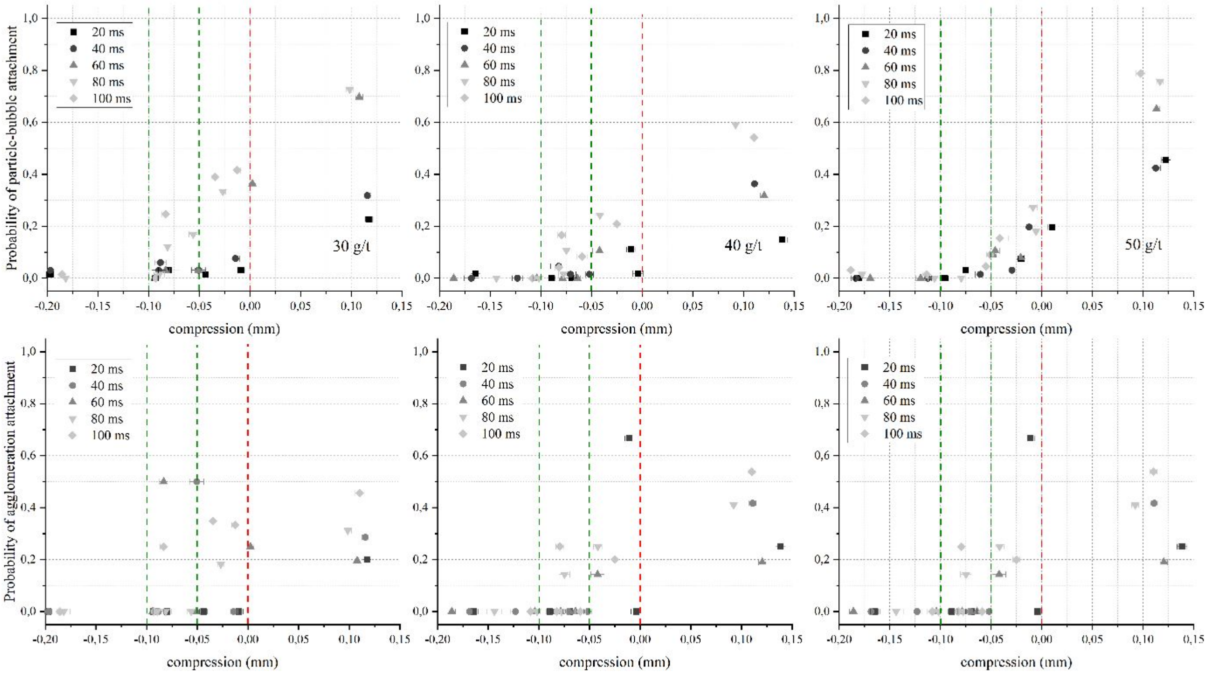

S3: Probability of particle-bubble and agglomerate attachment at 10 ppm DF250 and different HAC concentrations.

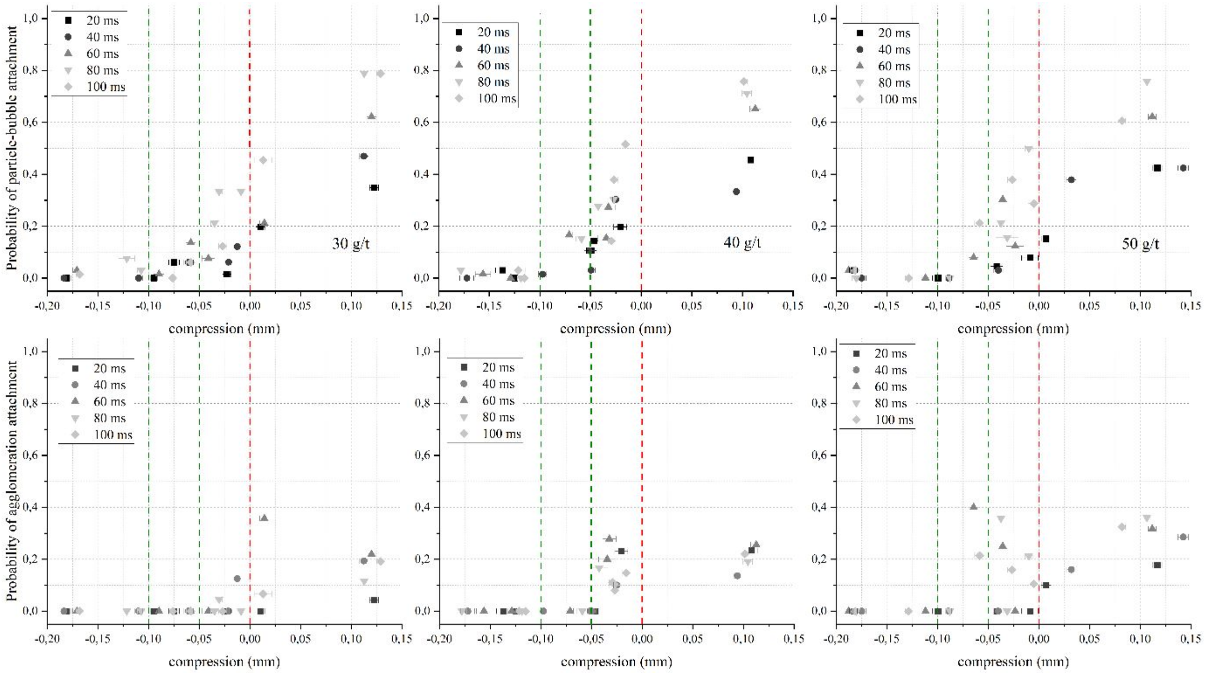

S4: Probability of particle-bubble and agglomerate attachment at 15 ppm DF250 and different HAC concentrations.
